# Evaluative study of a MOOC on knowledge translation in five French-speaking countries

**DOI:** 10.1371/journal.pone.0299923

**Published:** 2024-04-01

**Authors:** Romane Villemin, Christian Dagenais, Valéry Ridde

**Affiliations:** 1 Department of Psychology, Université du Quebec à Montréal, Montreal, Quebec, Canada; 2 Department of Psychology, Université de Montréal, Montreal, Quebec, Canada; 3 IRD, Inserm, Ceped, Université Paris Cité, Paris, France; 4 Institut de Santé et Développement, Université Cheikh Anta Diop, Dakar, Senegal; Al-Ahliyya Amman University, JORDAN

## Abstract

Over the past few years, the RENARD research team has observed a sharp increase in the need for knowledge translation (KT) training. Given the high demand, it has been nearly impossible for the team to provide training entirely in person, and so a massive open online course (MOOC) was developed. Its aim is to promote the use and appropriation of the KT process by practitioners, decision-makers, and others in the public sphere. The goal of this study was to evaluate the MOOC by collecting users’ opinions, reactions, appropriation, and practice changes. A qualitative research design was used. Data were collected through semi-structured individual interviews (n = 16) with professionals from Canada, France, and three West African countries (Burkina Faso, Mali, and Senegal) who had taken the MOOC. All interviews were subjected to thematic content analysis. The MOOC content was generally appreciated and reused by the respondents. The results revealed one main motive for completing the course: the immediate opportunity to apply their learning in their practice environments. However, most respondents deplored the lack of interaction among learners and expressed the need for coaching with an instructor to deepen the topics covered during the course. The results also revealed connection and accessibility issues linked to the Internet network and unstable access to electricity in West African countries. The study highlights the potential of MOOCs for the acquisition of knowledge and competencies by KT professionals. Several recommendations and avenues of exploration were formulated to optimize and improve future designs of MOOCs on KT.

## Introduction

Over the past few years, the RENARD research team has observed a sharp increase in the need for training in knowledge translation (KT), defined as “all the measures undertaken to make research activities and results known…. with a view to their use by practice settings, decision-makers, and the public, whether the approach is interactive or not” [1, p. 9]. Since 2009, our team based in Quebec (Canada) has been dedicated to KT research and training in the field of social interventions. We aim to advance both the science of KT and the KT practices of our partners. However, the demand for training is so great that our team lacks the availability to respond adequately with face-to-face training. Yet teaching the process and combining several KT strategies can facilitate the use of research-based evidence in practice and decision-making environments [2, 3] and thereby reduce the gap between the scientific knowledge produced and its use [4, 5]. Thus, it has become a priority to train researchers, political decision-makers, and stakeholders so they can work together to disseminate empirical data and provide access to best practices in their respective environments.

Given their ease of access and flexibility, the potential of online training courses for training professionals is well recognized [6–9]. The “massive open online course” (MOOC) format is one of the solutions available. MOOCs are distance learning courses offered online, accessible, and open to all. MOOCs make it possible to reach people who could not be reached in person due to lack of time or financial resources. For example, in less than two years, the RENARD team’s *Introduction to KT* MOOC has attracted almost 3,000 learners from 53 countries around the world. In the 1980s, with the advent of the Internet and the development of telematics, a digital era of distance learning, also known as the “digital campus”, was born [8]. Learners could now take courses at home and complete their learning online and by e-mail. Geographical distance between learners and teachers was no longer an obstacle [10]. There are several generations of distance learning [11]. They enable working people to access academic knowledge [12] and research-based evidence without having to enroll at university and therefore without having to pay tuition fees. MOOCs can be classified under several distinct models. In the literature, it is the cMOOC and xMOOC dichotomy that arises most frequently. xMOOCs are built by one or more experts and are reminiscent of the traditional classroom structure. In this model, participants are “consumers of knowledge” [13] with little interaction or freedom [14]. xMOOCs focus on knowledge transmission in the “experts to learners” direction. In contrast, cMOOCs focus on the participation of, and discussion among, learners, who together develop the course content. The decline in popularity of the original MOOC models in recent years has led to the development of variants such as hMOOCs [14–17], hybrid formats halfway between xMOOCs and cMOOCs. The challenges of MOOCs are linked to low completion rates, issues of intellectual property of pedagogical content, difficulties in verifying the identity of participants, as well as non-diploma and often fee-based certification [12, 14].

With a view to democratizing knowledge and making it accessible to as many people as possible, the RENARD research team, in collaboration with the Institut de Recherche pour le Développement (IRD) and the Centre de pédagogie universitaire (CPU) of the Université de Montréal (UdeM), has developed a series of asynchronous, self-supporting French-language online training courses. Their first MOOC, *Introduction to KT*, has been available since June 2020 via the EDUlib platform. This format was chosen because free access to quality education is the key to sustainable social and economic development, especially for people in remote areas.

While numerous studies have explored the potential of MOOCs, existing literature reveals certain gaps. Primarily, most studies have focused on comparing the effectiveness of MOOCs for knowledge acquisition with traditional face-to-face training [7]. However, for the advancement of best practices and the optimization of distance learning, a comprehensive understanding of the mechanisms contributing to user-perceived effectiveness or ineffectiveness is imperative. Secondly, there is a lack of qualitative studies aimed at understanding learners’ motivations, perceptions, and feelings about MOOCs [18–20]. Yet such information would enhance the effectiveness of future designs based on multicultural needs and user expectations. Moreover, the evaluation of MOOCs in the specific context of professional development [20] is underrepresented compared to online courses for students. Therefore, this study focuses on collecting data from a professional audience, excluding students. Additionally, the literature review did not uncover research dedicated to evaluating French-language MOOCs on KT. On the other hand, there are still few French-language online courses aimed at building capacity in KT. Furthermore, many people are still skeptical and question the pedagogical and educational value of MOOC [21]. By capturing learners’ reactions, this project will provide insights for future comprehensive evaluative studies on how MOOCs can effectively complement face-to-face training and contribute to the professional development of KT practitioners.

### Background to MOOC 1, introduction to knowledge translation

**Fig 1:**
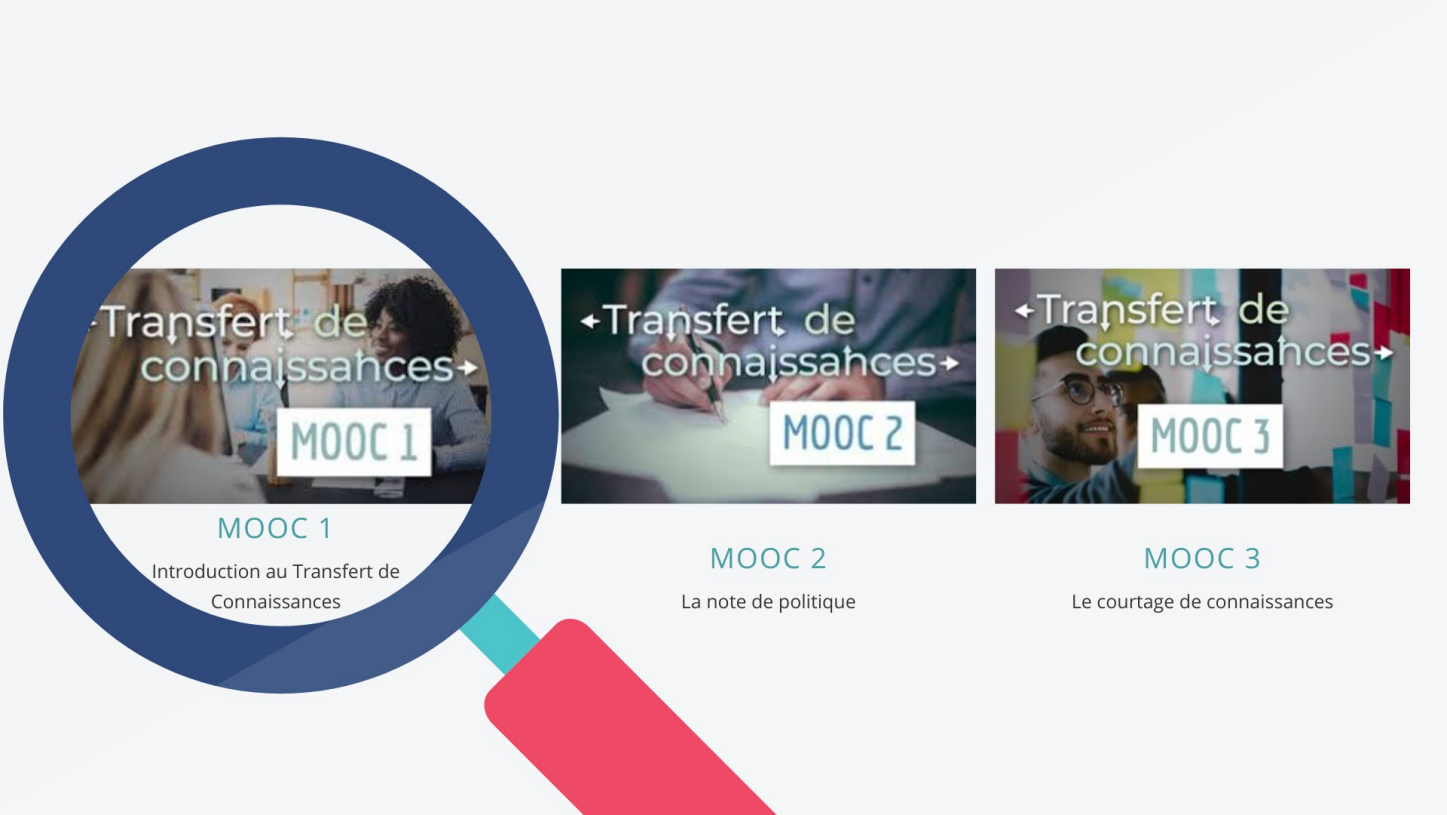
MOOC series on KT.

MOOC 1, *Introduction to KT*, is the first of a series of three courses (Fig 1) and is the one evaluated in this study. MOOC 2 deals with policy briefs, while MOOC 3 deals with knowledge brokering. All three MOOCs are based on the latest evidence in KT. There are no time limits or deadlines for registering and viewing content. This series is aimed at a specialized audience of producers and users of research data. It aims to encourage KT practices and the use of research evidence by practitioners and policymakers to optimize health policy decisions and interventions. The MOOCs are brought to the attention of the public through their dissemination on social networks (Twitter, Facebook). The RENARD team and its partners bring together hundreds of researchers in a dozen countries around the world, all of whom have been invited to participate and relay the information. The course can also be accessed via the RENARD team home page.

On September 16, 2021, 923 people out of 2,007 indicated at the start of MOOC 1 that they intended to complete all the proposed activities and potentially also request the attestation of completion. Four months later, as of January 17, 2022, 323 people had obtained the attestation, and all who had completed the course had requested it, for a completion rate of 35%.

The first MOOC introduces learners to basic concepts in KT (definitions, models, practices). The main KT activities and tools proven effective by research are also presented (policy briefs, knowledge brokering, deliberative workshops, infographics, planning a KT approach, and evaluating KT strategies). It offers massive, free access with no registration limits. Of the learners registered for MOOC 1 on May 21, 2022, 34.6% were from Africa, 32.1% from Canada, and 15.7% from France.

This course has four learning objectives: 1) to define the basic concepts of KT and use; 2) to identify KT mechanisms and activities; 3) to plan a KT process; and 4) to plan an evaluation of KT impact. Its overall aim is to encourage KT practices and the use of research evidence by practitioners and policymakers to optimize health policy decisions and interventions.

**Fig 2:**
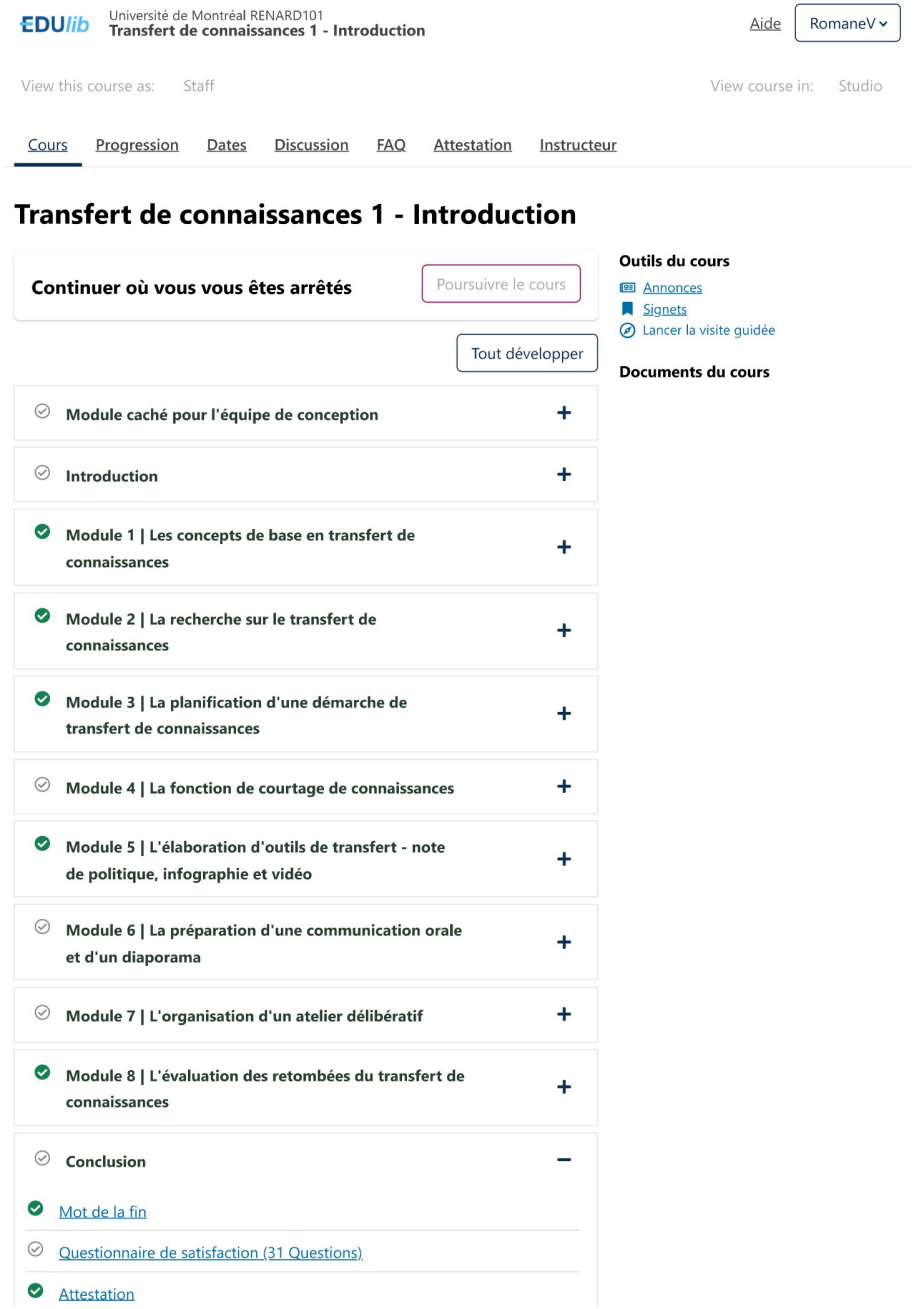
MOOC 1 table of contents.

It comprises eight modules (Fig 2): 1) basic concepts in KT; 2) the state of research on KT; 3) planning a KT approach; 4) the knowledge brokering function; 5) an overview of KT activities: policy brief, infographic, video; 6) an overview of KT activities: oral communication and slide show; 7) an overview of KT activities: deliberative workshop; and 8) evaluating the impact of KT. Each module is divided into three or four parts: 1) educational content; 2) experience sharing (for modules 1, 2, 3, 4, 5, and 7); 3) module review; and 4) module summary. In addition to the educational videos, several other activities are available, such as concrete examples, bibliographical references, training quizzes, Vox pop, summary sheets, and a practical exercise in module 6.

The summary sheets, which summarize the key concepts for each module, are downloadable once the module exam has been completed. The Vox pop capsules are divided into several parts. First, speakers, researchers, and policymakers from different countries and disciplines share their KT experiences of (presentations of a KT application, presentation of a KT research project, presentation, and feedback on the use of a KT strategy, etc.). They then answer one of the following questions: 1) What does KT mean to you? 2) What led you to KT? 3) What can KT do for your organization? 4) What factors influence research use? 5) What are your experiences of KT? and 6) What are the challenges associated with KT?

To adapt the course content to their partners and colleagues, the designers made a point of not focusing on Western examples, presenting studies carried out in Quebec as well as studies carried out in Africa, notably Burkina Faso.

## Methods

### Notion of completion

In this project, the concept of completion was examined by focusing on professionals whose original stated intention was to complete all course activities and possibly apply for the final attestation. While there are other definitions of the concept of completion [14, 22], in the context of this project the term refers to the professional’s completion of the MOOC course, with or without obtaining the final attestation. In other words, the learner persevered and completed all the modules to the end of the course and requested the attestation.

### Research design, sample, and recruitment

This study was based on an exploratory qualitative design to investigate facilitating factors and barriers to MOOC completion. The project focused on the perspectives of professionals from Canada, France, and three West African countries (Mali, Senegal, and Burkina Faso) who had taken MOOC 1 and whose initial stated intention was to complete it. This choice facilitated recruitment, leaving out a large proportion of “non-active” registrants who did not complete at least the first module of the course, as well as the “no-shows”, those who registered but never returned to the platform [21]. Choosing the countries with the most participants as of November 4, 2021, (Canada, France, Mali, Senegal, and Burkina Faso) provided a representative sample of views from countries with different histories and contexts.

With this in mind, and for the purposes of representativeness and triangulation of sources [23], interviews were conducted with individuals of all genders from countries with different professional, economic, and cultural realities.

Information collected in a questionnaire administered at the start of the course via the EDUlib platform was used to identify and sample the subjects who were then interviewed. The theoretical non-probability purposive sampling procedure was used [24]. This allows for a sample to be drawn that is not generated at random and is based on pre-established theoretical criteria, such as gender and profession. Thus, participants were “selected because their characteristics make it reasonable to expect that relevant information could be obtained efficiently" [24, p. 379], in line with the research question and objectives. Descriptive variables such as age, gender, country in which the course was taken, professional qualification, level of education, and other data such as reasons for enrolment and intentions regarding the MOOC provided a portrait of the learners.

### Qualitative data collection and analysis

After obtaining a certificate of ethical approval (CEREP-21-078-D) and the written consent of the interviewees, the individual semi-directed interviews were conducted and recorded via Zoom. The recruitment period for this study began on December 14, 2021, and ended on March 31, 2022. On average, interviews lasted between 30 and 40 minutes. This form of interview is the one most often used by researchers [25]. It provides access to respondents’ lived experience by structuring the production of their discourse while maintaining a certain openness to the emergence of new information and reflections [26]. The interview grid was created using concepts from the TIPEC (Technology, Individual, Pedagogy and Enabling Conditions) conceptual framework, a model designed to assess the quality of online training implementation. It is used to better understand the facilitators and barriers that affect e-learning success from the user’s point of view [27]. It comprises four categories of factors: technological, individual, pedagogical, and contextual. The potential impact of training on knowledge appropriation and application was also explored. The first part of the interview grid focused on individual and contextual factors (gender, job, area of expertise, organization and missions, country of MOOC delivery, knowledge of the KT process). The second part sought information on technological factors and MOOC accessibility (IT tool used, technical or technological difficulties encountered, appreciation of the EDUlib interface, forum). The third part aimed to obtain information on pedagogical factors and participants’ assessment of the content (adaptability of content, appreciation of the different activities presented in the MOOC). The final part explored their application of the knowledge and the potential impact of the MOOC (learning gained from it, usefulness of the training in practice). Three pilot interviews were conducted in March, April, and June 2021 to improve and adjust the interview grid before finalizing it. The learners’ research supervisor and the RENARD team’s research coordinator provided feedback and participated in the development and validation of the interview grid. Data were then collected from 16 learners who had completed the MOOC. Three learners preferred to respond in writing, due to lack of time or unstable Internet connection.

The final number of interviews was determined once theoretical data saturation had been reached. This principle involves collecting data until the interviews no longer yield any new information relevant to achieving the research objectives [26, 28, 29]. The number of interviews was therefore decided iteratively according to what emerged from the data.

All interviews were subjected to partial transcription, thematic content analysis [24], and mixed coding using NVivo software. Partial transcription speeds up the transcription process when the person coding is the same person who conducted the interviews. The procedure consists in transcribing and summarizing verbatims by time segment, without noting superfluous extracts that seem unimportant for answering the research question. For example, for the first few minutes of the interview, it’s common to transcribe “research presentation” without going into the details of what was mentioned verbatim. Researchers often use the third person singular or plural to begin interpreting interview data: “Emmanuelle mentioned that…and gave an example….” Also, if the person rambles in these explanations, or if there are silences, the researcher will not transcribe everything. For the purposes of this study, the first-person singular was preferred, as it seemed to better reflect the learners’ opinions when presenting the results. The NVivo software facilitates manipulation of the data and promotes rigorous transcription, coding, and verification. The aim of the thematic method is to highlight recurring themes, regularities, and discrepancies in the discourse [30]. Coding consists in labeling the verbatim extracts in such a way as to place discourse traces in categories [31]. These traces are “translated into the terms of a general, common, and more abstract language, specific to the researcher and no longer to the informants. This coding will enable the researcher to compare, classify, and synthesize what each informant has been able to contribute to his or her own language” [24, p. 7]. The closed side of coding is based on the categories and codes present in the TIPEC conceptual framework described above. The rest of the approach is more inductive (open coding). New codes, such as the notion of “accompaniment”, emerged from the data and enriched the analysis grid. Once data coding was complete, counter-coding (external validity) and reverse coding (internal validity) were carried out. Counter-coding establishes whether two researchers assign the same code to the same unit of meaning (inter-coder validity). This step made it possible to fine-tune the coding by, for example, merging the “contextual factors” code with the “individual factors” code. Indeed, their distinction was not clear, even in the lexicon. Reverse coding, on the other hand, involves checking the credibility, reliability, and consistency of the material [24] by recoding meaning units out of context (intra-coder validity). For example, it was decided to refine the “application of learning” code by dividing it into four sub-codes: 1) reuse of notetaking and downloads; 2) concrete examples outside of research; 3) concrete examples in research; and 4) no opportunity. These two verification stages led to a third and final coding grid.

## Results

The results are presented as follows: 1) sociodemographic portrait of the interviewees; 2) identification of facilitators and barriers leading to MOOC completion (TIPEC model); and 3) effects and potential impacts of the MOOC. As a reminder, the TIPEC conceptual framework is a model designed to assess the quality of online training implementation. It is used to better understand the facilitators and barriers that affect e-learning success from the user’s point of view [27]. It comprises four categories of factors: technological, individual, pedagogical, and contextual.

### Profile of interviewees

Interviews were conducted with 16 people who had completed the MOOC (Table 1) in the following countries: Canada (5/16), France (5/16), Burkina Faso (2/16), Mali (2/16), and Senegal (2/16). These people worked in the following fields: health (8/16), social services (2/16), education (2/16), engineering (2/16), administration (1/16), and international development (1/16). Interviewees included one transgender person (1/16), eight cisgender women (8/16), and seven cisgender men (7/16). All interviewees were French speaking. All respondents had a university education prior to their professional occupation, and half indicated that they already had some basic knowledge of KT before taking the course. The abbreviations F, and M in Table 1 stand for woman (8/16), man (7/16) and transgender man (1/16). The “1^st^ cycle” category corresponds to a bachelor’s degree in Canada or a license in France and Africa. The “2^nd^ cycle” category corresponds to a master’s degree in Canada or a Master II in France and Africa. The “3^rd^ cycle” category corresponds to a doctorate anywhere in the world. The abbreviations CA, FR, BF, ML, and SN stand for Canada, France, Burkina Faso, Mali, and Senegal, respectively.

**Table 1:** Profile of interviewees who completed MOOC 1 (n = 16).

Case	Country	Profession and area of practice	Type of organization	Gender	Age range	Education level
**1**	CA	Head of knowledge transfer and outreach, Department of Teaching and University Affairs (Administration)	University hospital	M	36–45	2^nd^ cycle
**2**	CA	CEGEP professor and researcher (Education)	Educational institution	F	46–55	3^rd^ cycle
**3**	CA	Engineer (Aeroportuary)	Airport	M	65+	2^nd^ cycle
**4**	CA	Director of a university library (Education)	University	M	56–65	2^nd^ cycle
**5**	CA	Project engineer (Livestock and animal production)	Not-for-profit organization	F	36–45	1^st^ cycle
**6**	FR	Teaching physician (Health)	University hospital	M	36–45	3^rd^ cycle
**7**	FR	Capacity building project manager (International development)	Research centre	F	25–35	2^nd^ cycle
**8**	FR	Program manager (Health)	Non-governmental organization	F	36–45	2^nd^ cycle
**9**	FR	Implementation manager (Health)	Research centre	F	36–45	3^rd^ cycle
**10**	FR	Documentalist (Health promotion)	Health education and promotion association	F	46–55	2^nd^ cycle
**11**	BF	Looking for a position (Health rights)	Research centre	F	25–35	3^rd^ cycle
**12**	BF	HIV call center manager (Social services)	Call centre	F	36–45	1^st^ cycle
**13**	ML	Communications officer (Social services)	Community-based organization	M	25–35	2^nd^ cycle
**14**	ML	Monitoring and evaluation manager (Health)	Non-governmental organization	H	25–35	2^nd^ cycle
**15**	SN	Research physician (Health)	Research centre	H	25–35	3^rd^ cycle
**16**	SN	Project manager (Healthcare)	Research centre	H	25–35	3^rd^ cycle

### Identification of facilitators and potential barriers to completion

In this section, the results are presented in terms of the TIPEC model [27].

#### Individual and contextual factors

*Motivations*. Most learners registered for the MOOC for professional reasons, in order to broaden their horizons in KT and to be able to apply the training content to their practice. One learner said that he would like to “contribute to the implementation of a KT program” as part of his work, with the aim of “raising awareness of the fact that a library must also support KT activities” (case 4). Another explained that this training had been suggested to him as part of his research project, because “at some point we’re going to have to pass on the knowledge we’ve gathered on the basis of the research, so that it’s useful to us right away in the field” (case 16). Several indicated that they wished to have a direct impact on policy decisions in the field of health in France (case 10) and in West Africa (cases 9, 15, 16). One learner explained that “researchers in the South are generally convinced [of the usefulness of KT], because in their countries things are so porous that they know the ministers, and so there’s more discussion between the two worlds” (case 7). Others took the course to gain doctoral credits (case 6) or credits for continuing education required by a professional order or employer (cases 3, 5, 10). Two learners took the course to develop their general knowledge and did not plan to apply the training directly to their jobs (cases 3, 13). Two wanted to “consolidate their knowledge of KT” (case 10) after attending practical workshops presented by the RENARD team: “the course enabled me to understand more about what (the trainer) had shown us in practice, and also to understand that there are other KT techniques apart from the policy brief” (case 15). One learner took the course as part of his job simply to update his theoretical knowledge of KT (case 1).

#### Openness to KT practice

Most learners attached great importance to the KT process. As one put it: “I get the feeling that it’s important. I find it frustrating that when a scientific publication comes out, the researcher is happy and moves on. Not only do most people not understand it, but it won’t be read. How do you expect to bring about change under these conditions?” (case 7). Overall, the learners were convinced that their workplace was open to KT culture and provided a favorable context for its use. Nonetheless, several mentioned barriers to the use of KT, including lack of knowledge about the KT process among some of their colleagues, lack of time, lack of support, and lack of available economic resources. As one learner put it: “It’s not yet fully part of our organizational culture, so it’s something we need to improve. We’re going to hold a webinar to make all our colleagues more aware of KT activities and to ensure this is done more systematically” (case 8).

#### Technological factors and accessibility

*Assessment of the EDUlib IT platform*. There were few comments about the MOOC interface. Overall, EDUlib seemed to have met learners’ expectations, and they were generally satisfied. All found the platform easy to use. Some mentioned the ideal length of the videos (“not too long”), while others appreciated the good sound and image quality. Five learners noted an ergonomic challenge linked to starting the course: “Even though I put the course in my favorites bar, each time I had trouble getting back to where I had been. Maybe we’re too much of a Netflix generation, where we’re used to opening up and returning to exactly the right place, but in the MOOC, you have to go back through the summary every time” (case 7).

*Technical and technological challenges*. Most learners said they never encountered any technical or technological difficulties, whether they took the course with a computer, tablet, or smartphone. However, four encountered connection and accessibility issues linked to the Internet network and unstable access to electricity in Senegal, Mali, and Burkina Faso. In the big cities, such connection and electricity access issues are becoming less of a problem, but when the learner is on a mission or at home in a more remote region, these issues are still very present, particularly when downloading videos and resources.

#### Pedagogical factors and content assessment

*Advantages and disadvantages of the MOOC*. According to the participants, the MOOC offered a number of advantages, such as its flexibility, with no fixed timetable or rhythm, its relatively short duration, the wide range of activities available, its free access to knowledge without having to travel, the possibility of stopping and returning later to continue the course, and the ability to reconsult its content at any time, even after the end of the course. According to several learners, the quality of the pedagogical content was an additional motivating factor for completing the course. Most would recommend or had already recommended the course to colleagues, partners, or relatives.

Nevertheless, four learners (cases 3, 9, 12, 16) used this type of device out of necessity and not because they really appreciated the MOOC format: “I prefer face-to-face courses, but I take online courses given my [limited] availability. It’s a plus, an alternative of sorts” (case 9). On the other hand, there were a number of disadvantages, such as the absence of any obligatory dimension to the course (“We tell ourselves, I can do it tomorrow or another day, there’s no reminder system” [case 6]); the lack of interaction with other learners (“We don’t have anyone to talk to when it’s over” [case 7]); the lack of interaction with a trainer (or “an instructor to answer our concerns and [correct our] misunderstandings” [case 12]); the loneliness felt in front of the screen; and the lack of practical exercises, feedback, and teamwork.

Some learners developed strategies to counter the lack of interaction sometimes expressed. Indeed, several said they had been able to discuss the course content with their colleagues (cases 2, 8, 16), since “doing it together is a motivating factor” (case 16), their husband (case 5), or even a member of the RENARD team (cases 2, 8, 15): “(the trainer) was there and we could talk to each other, so I didn’t feel the need to discuss things on a platform” (case 2). The fact that some of the learners interviewed had been accompanied by a KT trainer before (case 15) or after (cases 2 and 8) taking the MOOC was new data that emerged from the analyses, as that status had not been targeted by the study and did not figure in the recruitment. One took the MOOC to establish in a clearer and more theoretical way what he had done in practice with a KT trainer (case 15). These three learners (cases 2, 8, 15) appreciated the coaching and found it “very useful” (case 2) to deepen the notions seen in the course and to work on specific aspects of their projects, such as drawing up “a KT action plan” (case 8).

*Most popular aspects of the course*. Most learners spoke of their interest in obtaining the attestation. One attached it to his file as part of an internal competition within his organization (case 7). Another used it to validate doctoral credits (case 6) and others, as proof of continuing education (cases 3, 5, 10). Some appreciated the attestation, as “employers are very interested in KT, especially research institutes. For example, I used it in an interview with a research organization, and they told me that the RENARD team were like gods to them, because they were discovering KT, and they were really interested in what I had learned during the course” (case 11).

In the same vein, one learner said: “I landed this new job in an agency, and during the interview the question related to KT and capitalization came up. The evaluator was amazed at the KT process I’d been involved in. They asked questions about how it worked” (case 14).

Some learners had also added it to their curriculum vitae (cases 1, 9, 12, 14). Two said that, while it was not directly useful for their CV, it enabled them to defend their readiness to carry out KT activities in their organization: “I can tell people in management that I’ve taken this training. Right now, I’m in charge of a cultural security project, and this allows me to tell them that I didn’t embark on this approach without getting trained and finding out how to implement KT strategies” (case 2).

The summary sheets also met with unanimous approval. Many learners also appreciated the sharing of experiences in the Vox pop and the practical examples, “because they’re concrete, examples that reinforce understanding when you haven’t understood something” (case 9); that learner would have liked to have more concrete examples.

Assessment activities, such as training quizzes and end-of-module exams, were also much appreciated: “I preferred the exams for each module because they enabled me to check that I had acquired the competencies I was trying to learn. When I didn’t pass the training quizzes, I watched the videos again. I prefer to test my knowledge at the end of each module to make sure I’ve understood everything” (case 11).

*Less popular aspects of the course*. The various aspects presented in this section constitute avenues of improvement for future designs. Although the pedagogical content of the MOOC seemed to have met learners’ expectations, some wanted to learn in greater depth and thought that “this introductory course, for someone who wants to do KT activities, is not enough. It would have been relevant to go further and suggest that learners follow other programs and training courses to go into greater depth” (case 4). One person commented: “There’s one module that I didn’t find complete enough compared to the others, module 7 on the deliberative workshop. I wouldn’t remove it, but I would add to it. I was expecting to learn how to organize a deliberative workshop, and I was a bit disappointed” (case 11).

The MOOC focuses solely on research-based evidence, but several learners would have liked to delve deeper into the KT process for other types of evidence, such as experiential or contextual knowledge. “There are some projects where [the MOOC content] fits very well, because we have a whole research component, but we have other projects where it’s more operational implementation, and there we had a lot more discussions with my colleagues around ‘what do we consider to be knowledge on these projects where there’s no formal research?’ We had to do a lot of thinking to identify what knowledge was transferable on these projects” (case 8).

Some learners would also have appreciated seeing the concrete application of KT activities; for example: “present a policy brief and its more concrete spin-offs” (case 10), and “here’s the scientific article, such and such KT activities were carried out and here’s concretely what that changed, it became such and such a law, etc.” (case 7).

Several learners also felt that the MOOC reduced KT to the field of health: “I would have liked to see an example that wasn’t health-related, because researchers with expertise outside the health network don’t necessarily see themselves in this” (case 7).

Most learners raised the issue of the lack of interaction among learners and expressed their need for support. As one put it: “I missed being able to discuss questions about my project” (case 2). Some learners would also have liked more practical exercises and feedback. Furthermore, according to the learners, the MOOC’s biggest technical shortcoming was the discussion forum. One learner suggested: “There should be a forum where we can ask questions and get the answer quickly” (case 16); and another: "We need to find these spaces where we can expand on KT and be able to discuss it” (case 7). As this MOOC is an introductory course, some learners did not feel any need to interact with an instructor or other learners; however, “if we were to go further into the course, we’d need the opportunity to interact, but for an introduction to the subject, interaction isn’t necessary” (case 1). With a few exceptions (2/16), bibliographical references were clearly not a priority for the learners interviewed; they took too long to consult, there were too many, or in some cases they were of no interest, given that “I’m not a researcher” (case 7).

Finally, several learners said the publicity for the course and the MOOC series in the broadest sense could be better: “The communication would need to be a little sexier…. the marketing aspect is important to create interest, even if research prefers to keep its distance from that” (case 7). One learner suggested: “For the series, we should develop automatic mailings by contacting those who have completed the MOOC to let them know a new MOOC is available. I was, in fact, just wondering whether the MOOC on knowledge brokering was ready, but I must check the site myself all the time” (case 4).

*Evaluation of the potential effects of the MOOC and its application*. In this section, we identify the course’s potential effects by conducting an exploratory assessment of the training’s effectiveness from the learners’ point of view. Of the learners who had completed the course, two did not plan to apply its content in their practice (cases 3, 13).

*Reuse of notes and downloads*. Several extracts from the interviews showed that the rest of the learners had used or planned to use the theoretical knowledge taught in the MOOC. Most had taken notes and downloaded the summary sheets and certain bibliographical references with a view to reusing them later. Some had already reused them: “[for my projects] I referred to the summary documents and my notes on policy briefs and the section on infographics. I’ve also reused them to explain to my colleagues what KT is and what it means for a project to do KT” (case 8).

*Perceived changes in ways of doing things and concrete examples in research*. Other learners had used the theoretical knowledge in their research assignments: “I had to write two policy briefs using what I learned in the MOOC” (case 11); “Right now we’re preparing a research grant for a project on prison education. The KT component is now something I want to put into our projects every time” (case 2). Another learner said: “I had to review my organization’s KT and outreach service offer in the fall, so I applied everything I’d learned. I got some ideas from the tools presented in the course, and we revised the way we presented the service offering, based on my updated knowledge of KT” (case 1).

One learner spoke of changes in the way he saw his target audiences: “Definitely, the main thing that’s changed is the way we see things, especially decision-makers who aren’t in research. The way I see them in relation to what I must tell them. It always makes me think ‘these people aren’t necessarily in your field, so you have to pay attention to what you want to tell them.’ It’s something I’ve kept in mind since the course” (case 14).

A few people also mentioned changes in their way of communicating: “I’ve learned to speak slowly and less, to choose my words carefully, and to prepare my presentation in advance so as to keep people with me” (case 11).

Others spoke about changes in the vocabulary used: “The most obvious thing that has changed has to do with vocabulary. Before, I would have talked about appropriation. Mostly, before, it was an implicit activity, but now we can write the term KT, and that means it’s something that can be funded and recognized. It’s becoming an activity in its own right, a well-founded activity and no longer a matter of intuition” (case 10).

Finally, others described changes in their written PowerPoint communications: “When I took part in two deliberative workshops, I was able to adapt the content that I had to present. The notion that has stayed with me is ‘one slide, one idea.’ Initially, there were too many ideas in my presentations” (case 11).

*Perceived changes in ways of doing things and concrete examples outside of research*. Even outside of research, some learners were able to apply what they had learned during the MOOC. They followed the advice given in the modules to assimilate it and transpose it to their reality: “Here at headquarters, they make two-page summary sheets on a subject related to sustainable development and interdisciplinarity. They asked me to do one and to present it, and so I applied the advice given for policy briefs” (case 7); “I think KT should systematically be part of our thinking on all our projects, whether or not they have a research dimension, to reflect on what we generate in terms of knowledge or learning, and then to think about what we want to transfer, to whom, and when” (case 8).

To sum up, the topic that came up frequently was the acquisition of tools and methodologies.

## Discussion

The aim of this study was to evaluate MOOC 1, *Introduction to KT*, developed by the RENARD team. The results showed that this MOOC was generally appreciated by respondents. They appreciated its flexibility, with no set schedule or pace, its relatively short duration, the wide range of activities available, the fact that certification was free, the quality of the content presented, the accessibility of knowledge without having to travel, the possibility of stopping and returning later to continue the course, and the fact that the content could be reconsulted at any time, even after the course had ended. Nevertheless, the most cited areas for improvement were the lack of interaction, of concrete examples outside the healthcare field, of presentation of the KT process for other types of evidence, and of effective publicity. For some, the MOOC also seemed to have had an impact on their knowledge use and inspired changes in practice. While the results cannot be generalized to the entire MOOC population, they provide interesting insights from which a synthesis of MOOC facilitators and barriers can be drawn (Table 2), as well as hypotheses and recommendations.

**Table 2:** Summary of the main facilitators and barriers of MOOC 1.

Categories	Facilitators	Barriers
**Contextual and individual factors**	Prospect of direct application in practice	Learners’ lack of openness to new technologies
	Learners’ openness to new technologies	Having other professional priorities
**Technological factors and accessibility**	No technological difficulties encountered	Challenges related to Internet connection and access to electricity
		Unappealing visual interface
**Pedagogical factors and content assessment**	Free certification	Lack of interaction with an instructor
	Good quality content	Lack of interaction with other learners
	Exams for each module	
	Wide range of activities	
		Teaching content not adapted to certain participants
		Lack of practical exercises and feedback

### Potential for immediate application in practice

There was a wide variety of cultures and profiles represented among the “MOOCers” of the *Introduction to KT* course. However, this study showed again that the people who sign up for the MOOC are generally graduates, who do so mainly with a view to acquiring knowledge and competencies useful to their professional missions [32]. This is what Carré and Caspar [33] call “professional operational motives.” An analysis of the results suggests, in fact, that “professional operational motivation” may be one of the most important individual factors leading to MOOC completion. The possibility of immediately applying the knowledge learned to their practice seemed to be the main motivation for the learners who had completed or planned to use the course. This must be borne in mind because, as several learners mentioned, the proliferation of training media has led an almost overwhelming embarrassment of choice. Moreover, the course is designed for an audience that is already well-informed and advanced in the field of KT. Thus, the course’s intentions need to be clearly explained and defined as soon as it is disseminated. It is important to clarify that the *Introduction to KT* MOOC is a course that deals only with research-based evidence and not with other types of knowledge, such as tacit knowledge. This clarification could reduce the number of registrations, but also the number of dropouts among individuals who see no direct link with their profession.

### Importance of the interaction dimension

The MOOC strategy has potential and could prove advantageous for the acquisition of KT knowledge and competencies. However, regarding competencies, the current model would need to be modified, as the *Introduction to KT* MOOC offers no possibility for discussion among participants or with an instructor. Despite the presence of a forum, the MOOC has not succeeded in creating spaces for dialogue. Yet in recent scientific literature, hMOOCs seem to be emerging as the ideal model that integrates the best of both worlds between xMOOCs and cMOOCs [14–17]. This hMOOC format may be an avenue worth exploring, as it retains the advantages of xMOOCs while, for example, giving participants the opportunity to interact with other learning professionals via social networks. Moreover, the “expert-to-learner” model (xMOOC) may be suitable for a course that introduces the subject matter, as in a lecture course, but for the application of KT strategies, the simple availability of knowledge does not appear to be enough. Future MOOCs could expand into a more collaborative approach to have an even greater impact on learners’ appropriation and practical use of knowledge. Some researchers, for example, suggest using social networks such as Twitter, a Facebook page dedicated to the course, or synchronous meetings to connect professionals with each other [17]. This is, however, a costly solution, as it requires active trainers to manage the social networks and facilitate interactions among learners. Indeed, as of August 25, 2022, MOOC 1 had reached 2,811 learners, and organizations offering KT training do not necessarily have the human and financial resources to interact individually with each learner, which is why the course is intended to be as self-supporting as possible.

### Other avenues for improvement

Based on the analysis of the interviews, points for improvement are presented in Table 3.

**Table 3:** Strengths and suggestions for improvement.

What works	Points to explore
Video length	Develop the interaction aspect by exploring the potential of more collaborative MOOC formats (cMOOC, hMOOC) and social networks
Exams for each module	Include personalized messages for participant follow-up
Wide range of activities	Add more concrete examples by extending to areas other than healthcare
Free certification	Put more effort into disseminating the course
	Offer a textbook-style learning aid that brings together all the knowledge from the MOOC as an alternative for people who have difficulty with new technologies or connection difficulties

### Study strengths and limitations

To date, few qualitative studies have assessed the quality and effectiveness of MOOCs in the African context. Yet such information would enable future MOOC designs to be modified and adapted to multicultural needs and user expectations. In most MOOCs, African countries are under-represented [32], but in this MOOC the reverse was true. Hence the importance of evaluating this course from the point of view of African learners, even if fewer of them responded to the recruitment calls for this project. A review of the literature to date has failed to identify any research devoted to the evaluation of French-language MOOCs on KT. Moreover, many people are still skeptical and question the pedagogical and educational value of MOOC devices [12, 24]. Hence the relevance of an evaluation of this introductory MOOC on KT. Because the method used was purely qualitative and the sample was limited to 16 respondents whose initial stated intention was to complete the course, the results obtained are exploratory and cannot be generalized to all learners. Moreover, given that all the learners interviewed had completed the course, it was not possible to illustrate all the barriers related to this MOOC. Also, the inclusion of learners who had been supported by a KT trainer before or after doing the MOOC (emerging data in the results), while not specifically targeted in the recruitment, could have biased the results. Nevertheless, the analysis of the discourse of these three learners appeared to reinforce the conclusions drawn about the interaction needs expressed by the other respondents. Also, due to a variety of difficulties encountered in recruiting learners in Africa, it was not possible to interview more than two people in each of the three countries. The phenomenon of social desirability may also be a limitation of this study. Indeed, some learners may have embellished their answers, since several worked for organizations that were partners of the RENARD Team. Finally, the first interviews began more than a year after the course opened, and some learners had little recollection of the MOOC’s activities.

## Conclusion

The evaluation results are positive. Completion of the MOOC appears to lead to the assimilation of knowledge and some changes in evidence-based practice. Respondents reported gaining vocabulary, tools, and methodologies because of the training. They appreciated its flexibility without set schedules or imposed pace, its relatively short duration, the variety of available activities, the free certification, the quality of presented content, accessibility to knowledge without the need for travel, the option to pause and return later to continue the course, and the ability to review its content at any time, even after completion. However, the lack of interactions, the absence of concrete examples outside the health domain, the lack of content about contextual, experiential knowledge, and ineffective advertising were the most cited areas for improvement by respondents. These results underscore the need to evaluate MOOC-type training, as even though the project was limited to interviewing learners who completed the course, they still highlighted potential areas for improvement to consider.

Despite the few adjustments and areas for improvement mentioned, this research shows that the content of the *Introduction to KT* MOOC was generally appreciated and reused by the respondents. The chosen MOOC strategy therefore has potential for the acquisition of KT knowledge and competencies. To gain a more comprehensive understanding of the phenomenon studied, future studies should deepen the research by using a mixed design that would include quantitative indicators in addition to the qualitative data collected. Pre- and post-MOOC evaluations would also be needed to assess more fully what was learned during the course. Finally, in addition to the evaluation of MOOCs undertaken independently, other studies should assess the added value of coaching while completing MOOCs on KT.

## Supporting information

S1 Appendix Interview grid. (DOCX)
