# Supplementary material for: Evaluative study of a MOOC on knowledge translation in five French-speaking countries
Source: PLoS One. 2024 Apr 1;19(4):e0299923. doi: 10.1371/journal.pone.0299923 (PMC10984463; doi:10.1371/journal.pone.0299923)
Supplement: S1 Appendix — (DOCX) [file pone.0299923.s001.docx]

**INTERVIEW GRID (completion)**

Evaluation of the MOOC on knowledge translation

Before we begin the interview and recording, I'd like to quickly introduce myself. My name is RV. I'm doing a master's degree in psychology at the Université de Montréal under the supervision of Professor CD. My thesis project involves evaluating the effectiveness and quality of the MOOCs on KT developed by the RENARD research team. Thus, your participation is solicited to evaluate the content, structure, progress, and potential effects of the MOOC on your practices.

I'm going to ask you around forty questions, divided into 4 sections.

The recording will be kept securely by me, and only members of the research team will have access to its contents. Do you agree to the interview being recorded?

During the interview period, don't hesitate to expand on your answers, even if it's outside the scope of the question.

The interview is likely to last between 45 and 60 minutes.

***Do you have any questions before we get started?***

***I'm going to start with some questions that will allow me to get to know you and the context in which you've taken the MOOCs.***

# Contextualization/contextual factors

1. Where did you access the course (country)?
2. What was the last diploma you obtained?
3. What job/position do you currently hold?
4. Could you briefly describe your organization and its missions?
5. How important is knowledge translation in your work? Why or why not?
   - *Does this have any bearing on your professional activities?*
   - *Do you think that developing your KT skills is useful for you? In what way*?

# For those who have requested a certificate of completion:

- - How did you use this certificate?

# Why did you take this course? What were your motivations?

1. A slightly different question now, what do you think helped you make it to the end of the course?

***Now I'm going to ask you some related to the technology and accessibility of the course.***

# Technological factors/accessibility

1. What did you use to access the course? (Laptop, tablet...)
   - *Would you prefer to have access via your smartphone?*
   - *Was the content compatible with the IT tool you used? For example, was your computer or its system recent enough to host the platform?*
2. Did you encounter any difficulties connecting? Or any other technical/technological difficulties?
   - *Do you have a good Internet connection?*
   - *Has this affected your experience?*
3. Did you find the course interface user-friendly? What would you change (font size, colors, video/image quality)?
   - *Was the course structure well organized and easy to follow? If not, what would you change?*
4. Did you use the discussion forum? Was it relevant? Why or why not?

# I'm going to ask you some questions that focus on the content of the course.

**Pedagogical factors and content assessment**

1. Based on your lifestyle/values/culture, your job, does the course lack personalization/adaptability of content? Did it offend your values?
2. Do the subjects correspond closely enough to your reality?
   - *Were there any examples you didn't like? Which ones?*
   - *Is the language appropriate?*
3. What did you think of the exams? Were they too difficult?
4. Was the general content relevant? What would you add?
5. Do you plan to refer to and re-use the notes, resources and summary sheets provided during the course?
   - Do you still use the materials provided (summary sheets, readings) or do you revisit the platform if necessary?
6. Was there enough variety in the materials provided? Quality?
   - Which activity(ies) did you prefer (educational videos, quizzes, readings, practical exercises, Vox pop, summary sheets)? Why?
   - Which activity(ies) did you enjoy least? Why?
7. What do you think of the references/resources/readings available to you (quantity/quality)?
   - Have you read them? (All, most, just a few...)
8. What do you think would be the best way to get professional learners involved in such activities?

# Now I'm going to ask you some knowledge-based questions. We've reached the 20^th^

**question (half).**

# Individual factors

1. Did you already have basic knowledge of the course notions (in KT)?
2. Do you feel you lack computer skills? For example, are you able to solve problems or perform basic tasks?
3. Do you enjoy taking online courses? Why or why not?
4. Did you find the course easy to use?
5. Was the asynchronous mode relevant? Or did you feel a lack of interaction?
   - With an instructor
   - With other learners
6. Do you find this MOOC-type course format attractive? Why or why not?
7. In your opinion, what does an online course like this offer (*or not) compared to* a face-to-face course?
   - If you were to make a comparison between the two (*face-to-face versus distance learning*), what would be the advantages of the MOOC?
   - What are the disadvantages of the MOOC?
8. Has taking this course put any constraints on your schedule? How did you organize your time (work/family/study)?
   - During the e-learning sessions, were you in a supportive, distraction-free environment?
   - Did you have the support of your family and friends or your employer?
9. What are your expectations for a future course like this?

# I'll move on to questions about the potential impact the course has had on your practices.

**Application of knowledge and potential impact of the MOOC**

1. Do you have or plan to apply any of what you've learned in the course? Which ones?
   - How can I help? What do you think will be most useful to you? Why or why not?
2. How do you put what you've learned to **practical** use? Could you **give** me **an example of a KT application**?
3. Have any of your practices changed?
   - Can you give me a concrete example of a change in your practices?
   - How has this changed the way you do business?
4. Has anything changed in the way you communicate?
   - Can you give me a concrete example related to your practice?
5. What are the barriers to changing KT practices?
6. Are the people around you (researchers, people you work with) open to KT?
7. Would you recommend this course to others? Why or why not?

# Other

1. Do you have any other comments? Is there anything else you'd like to talk about?
   - What do you think could be done to improve this course?

Thank you for your cooperation!
